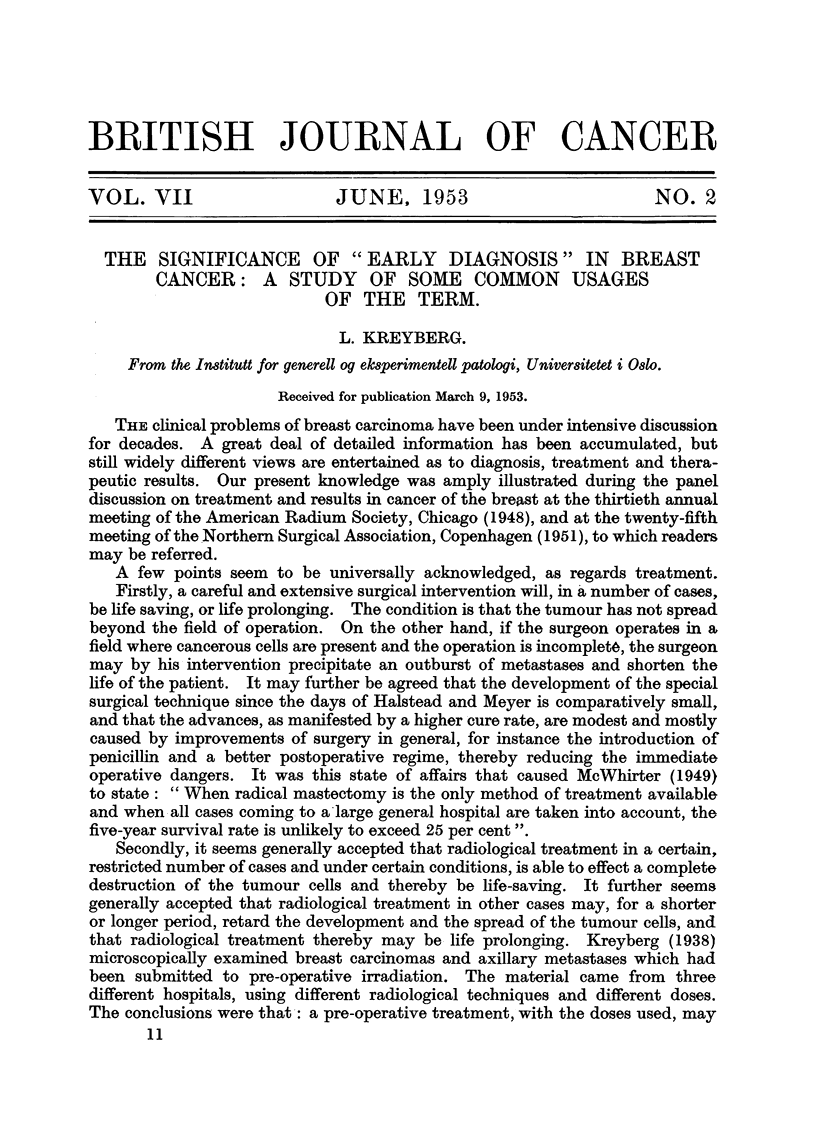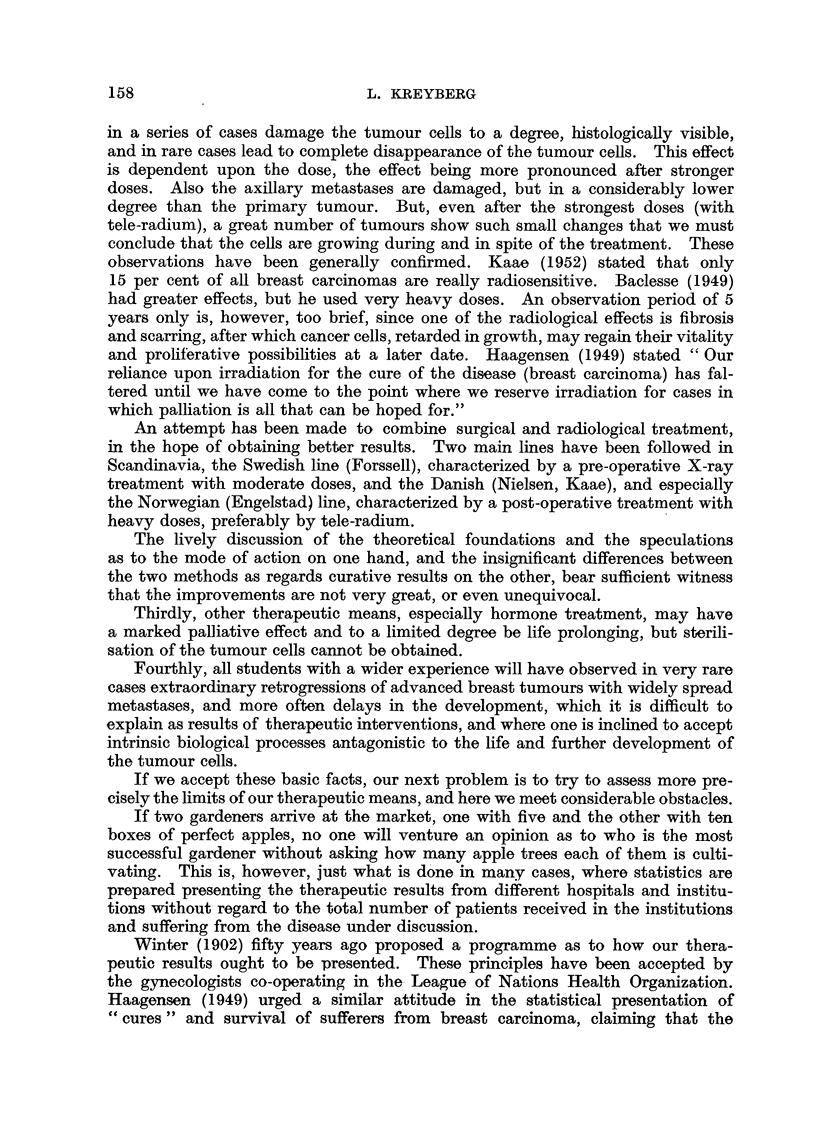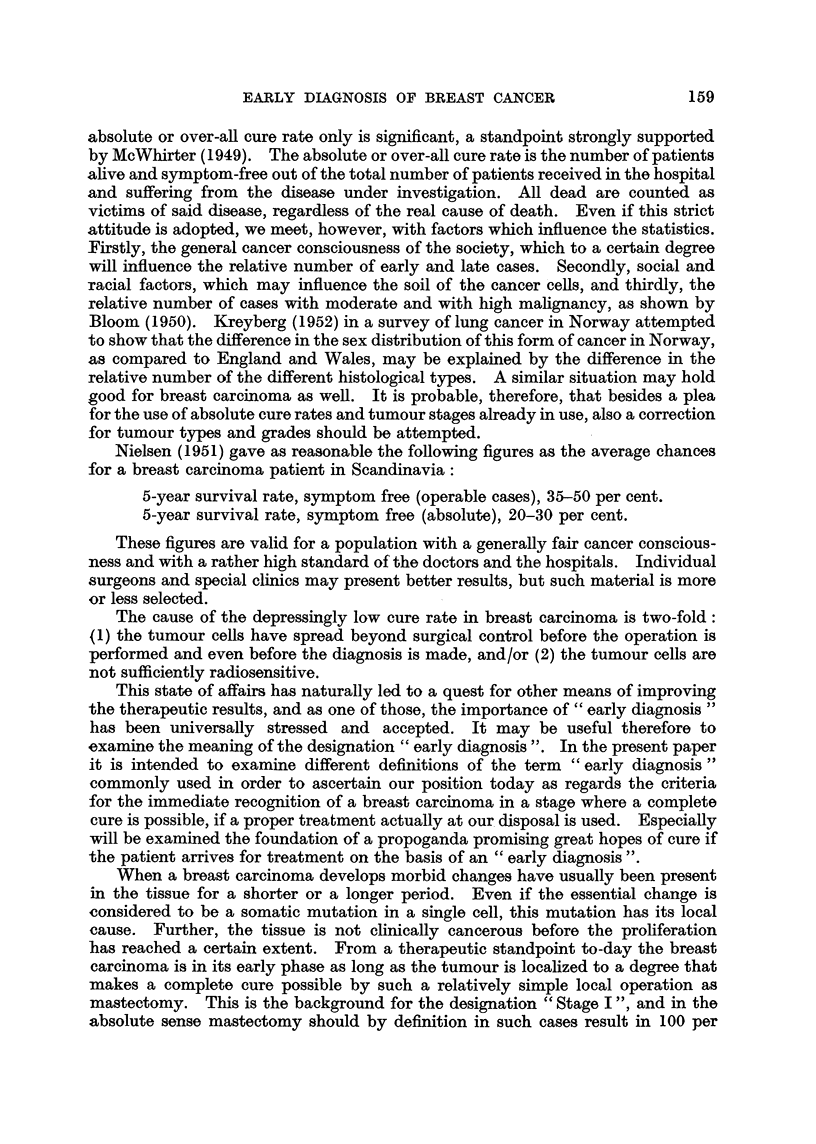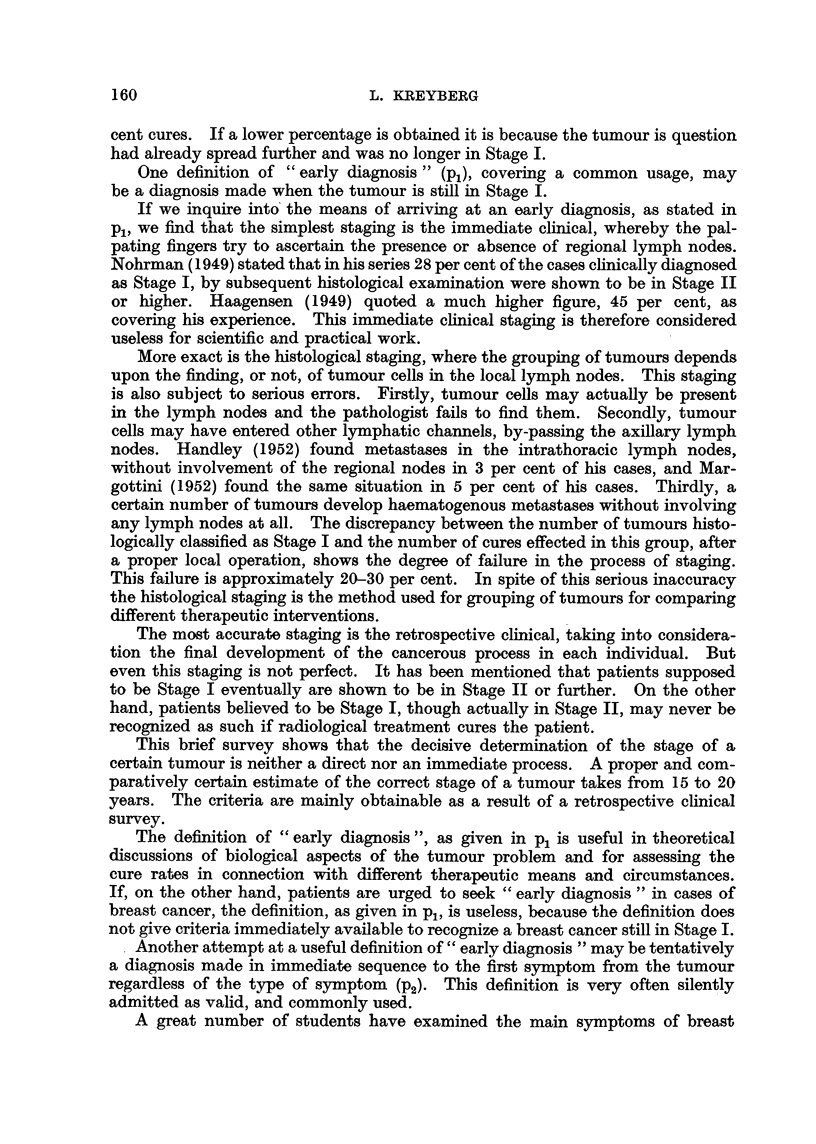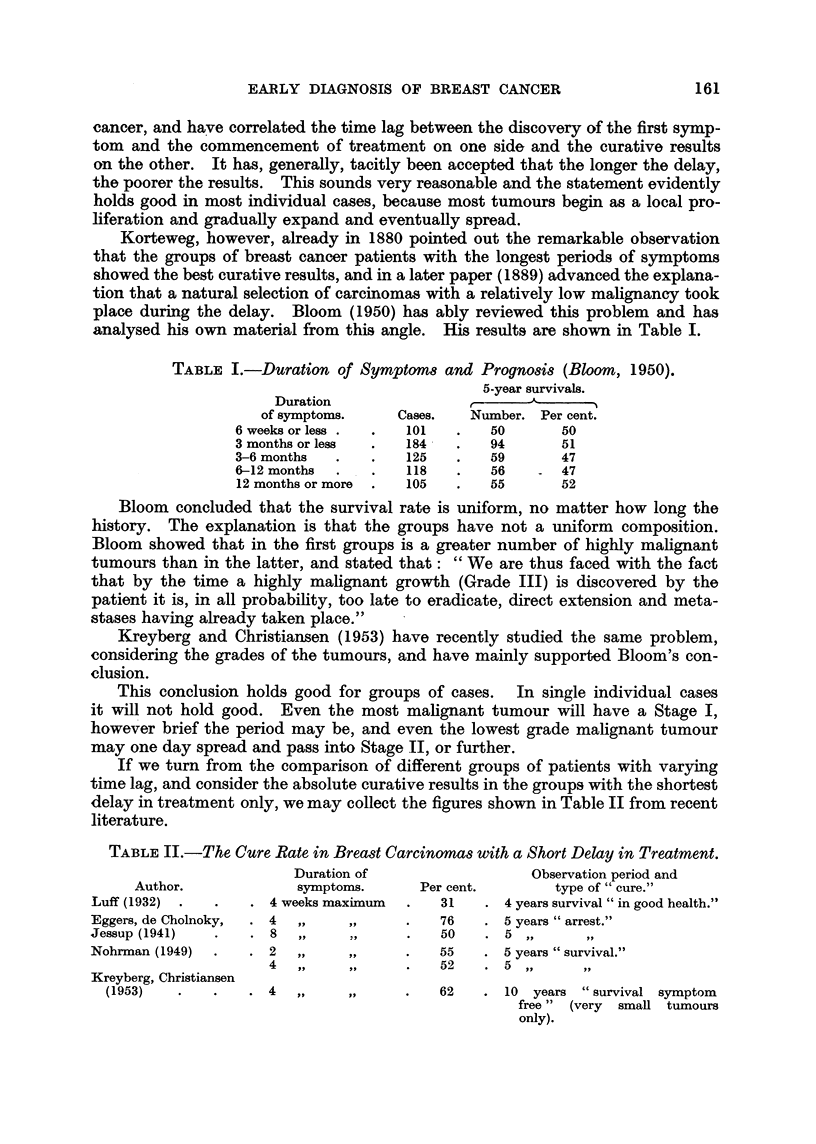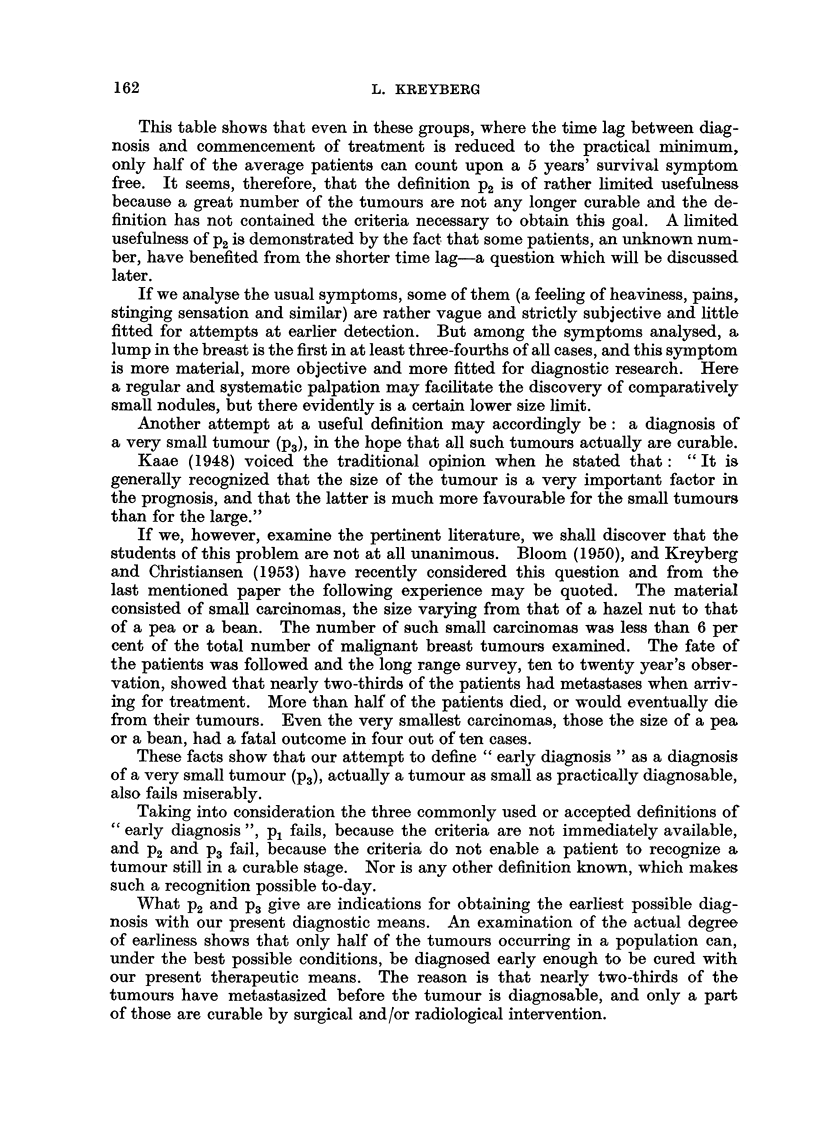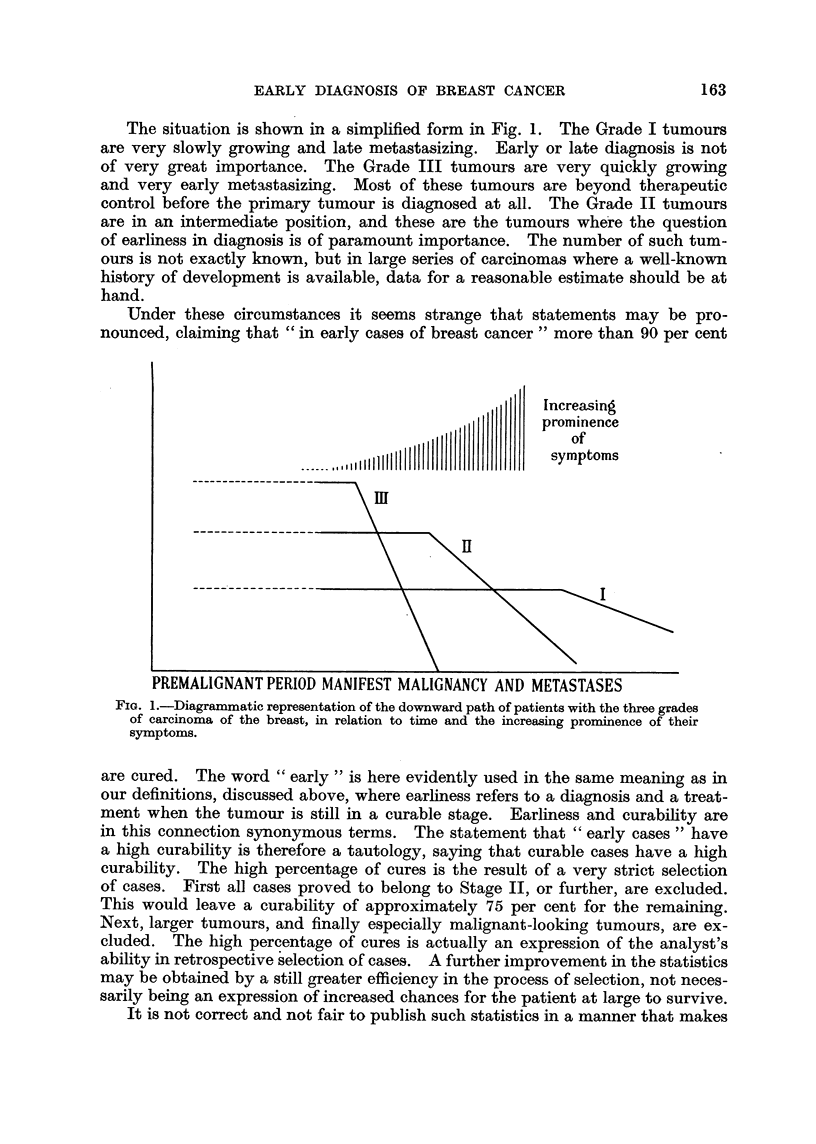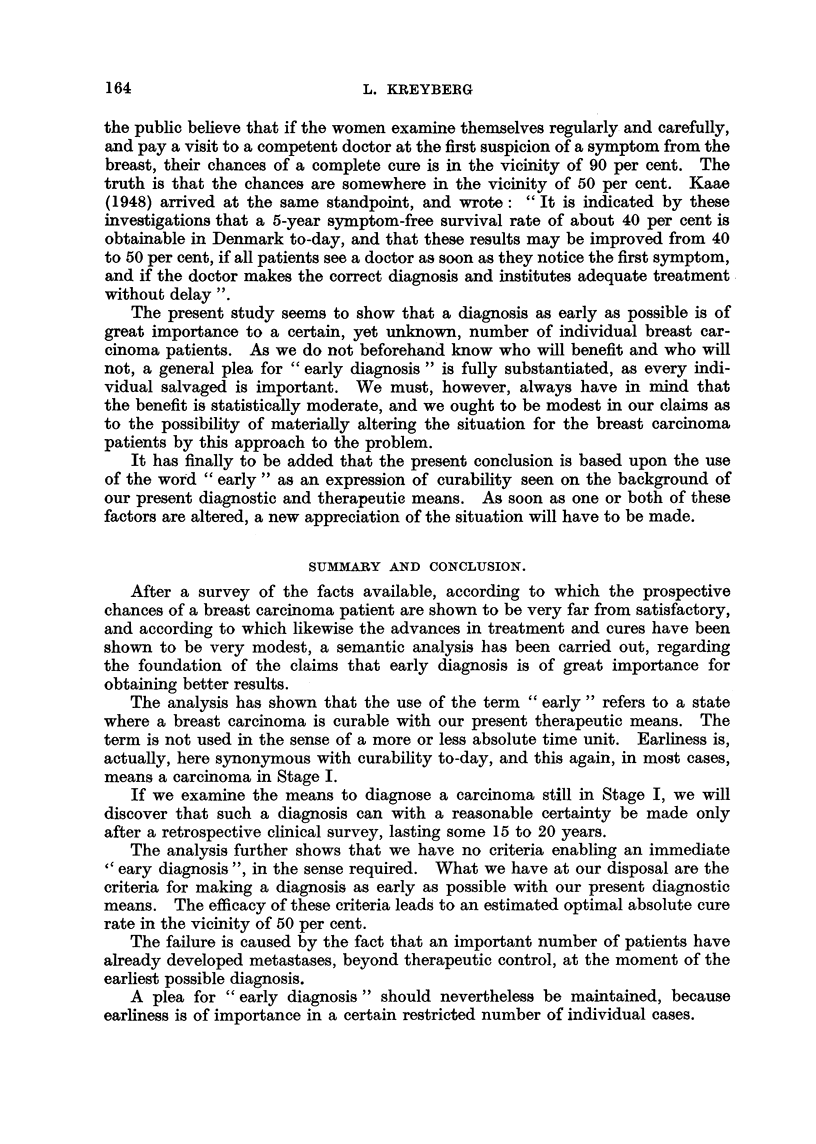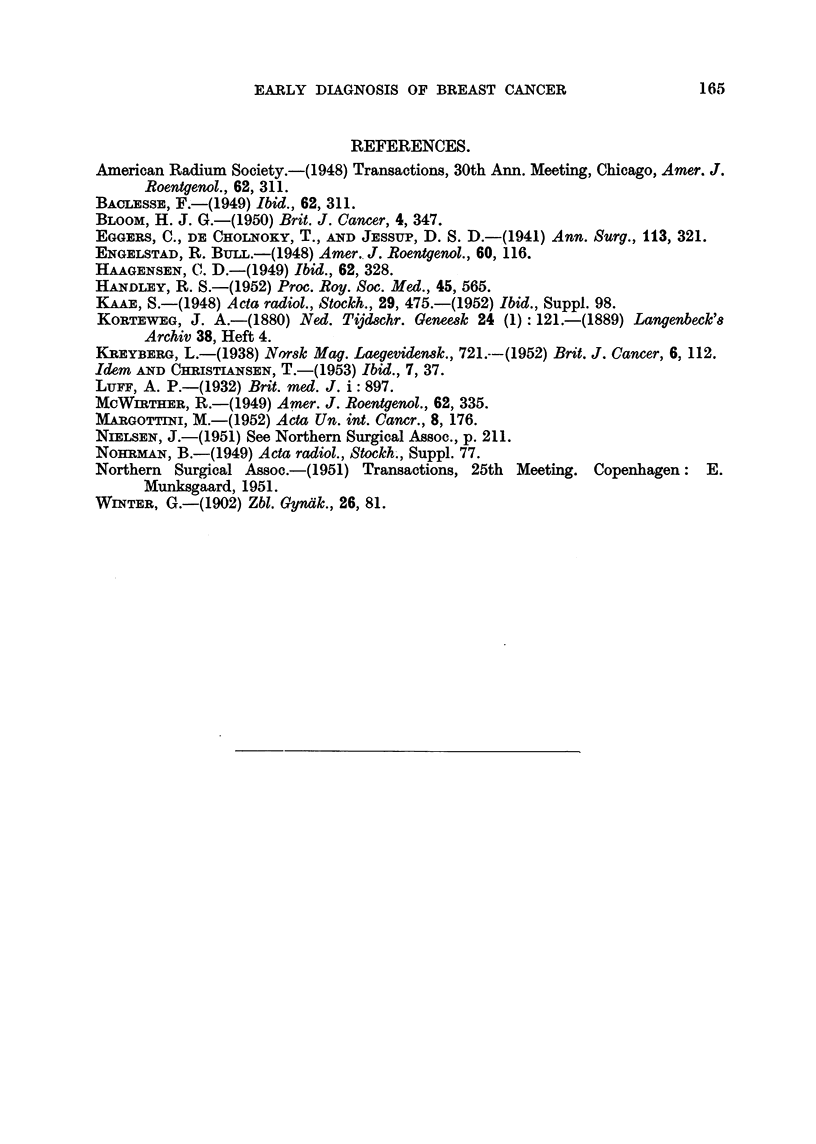# The Significance of “Early Diagnosis” in Breast Cancer: A Study of Some Common Usages of the Term

**DOI:** 10.1038/bjc.1953.15

**Published:** 1953-06

**Authors:** L. Kreyberg


BRITISH JOURNAL OF CANCER

VOL. VII JUNE, 1953 NO. 2

### THE SIGNIFICANCE OF "EARLY DIAGNOSIS" IN BREAST CANCER: A STUDY OF SOME COMMON USAGES OF THE TERM.

L. KREYBERG.

*From the Institutt for generell og eksperimentell patologi, Universitetet i Oslo.*

Received for publication March 9, 1953.

THE clinical problems of breast carcinoma have been under intensive discussion for decades. A great deal of detailed information has been accumulated, but still widely different views are entertained as to diagnosis, treatment and therapeutic results. Our present knowledge was amply illustrated during the panel discussion on treatment and results in cancer of the breast at the thirtieth annual meeting of the American Radium Society, Chicago (1948), and at the twenty-fifth meeting of the Northern Surgical Association, Copenhagen (1951), to which readers may be referred.

A few points seem to be universally acknowledged, as regards treatment.

Firstly, a careful and extensive surgical intervention will, in a number of cases, be life saving, or life prolonging. The condition is that the tumour has not spread beyond the field of operation. On the other hand, if the surgeon operates in a field where cancerous cells are present and the operation is incomplete, the surgeon may by his intervention precipitate an outburst of metastases and shorten the life of the patient. It may further be agreed that the development of the special surgical technique since the days of Halstead and Meyer is comparatively small, and that the advances, as manifested by a higher cure rate, are modest and mostly caused by improvements of surgery in general, for instance the introduction of penicillin and a better postoperative regime, thereby reducing the immediate operative dangers. It was this state of affairs that caused McWhirter (1949) to state : " When radical mastectomy is the only method of treatment available and when all cases coming to a large general hospital are taken into account, the five-year survival rate is unlikely to exceed 25 per cent ".

Secondly, it seems generally accepted that radiological treatment in a certain, restricted number of cases and under certain conditions, is able to effect a complete destruction of the tumour cells and thereby be life-saving. It further seems generally accepted that radiological treatment in other cases may, for a shorter or longer period, retard the development and the spread of the tumour cells, and that radiological treatment thereby may be life prolonging. Kreyberg (1938) microscopically examined breast carcinomas and axillary metastases which had been submitted to pre-operative irradiation. The material came from three different hospitals, using different radiological techniques and different doses. The conclusions were that : a pre-operative treatment, with the doses used, may in a series of cases damage the tumour cells to a degree, histologically visible, and in rare cases lead to complete disappearance of the tumour cells. This effect is dependent upon the dose, the effect being more pronounced after stronger doses. Also the axillary metastases are damaged, but in a considerably lower degree than the primary tumour. But, even after the strongest doses (with tele-radium), a great number of tumours show such small changes that we must conclude that the cells are growing during and in spite of the treatment. These observations have been generally confirmed. Kaae (1952) stated that only 15 per cent of all breast carcinomas are really radiosensitive. Baclesse (1949) had greater effects, but he used very heavy doses. An observation period of 5 years only is, however, too brief, since one of the radiological effects is fibrosis and scarring, after which cancer cells, retarded in growth, may regain their vitality and proliferative possibilities at a later date. Haagensen (1949) stated " Our reliance upon irradiation for the cure of the disease (breast carcinoma) has faltered until we have come to the point where we reserve irradiation for cases in which palliation is all that can be hoped for."

An attempt has been made to combine surgical and radiological treatment, in the hope of obtaining better results. Two main lines have been followed in Scandinavia, the Swedish line (Forssell), characterized by a pre-operative X-ray treatment with moderate doses, and the Danish (Nielsen, Kaae), and especially the Norwegian (Engelstad) line, characterized by a post-operative treatment with heavy doses, preferably by tele-radium.

The lively discussion of the theoretical foundations and the speculations as to the mode of action on one hand, and the insignificant differences between the two methods as regards curative results on the other, bear sufficient witness that the improvements are not very great, or even unequivocal.

Thirdly, other therapeutic means, especially hormone treatment, may have a marked palliative effect and to a limited degree be life prolonging, but sterilisation of the tumour cells cannot be obtained.

Fourthly, all students with a wider experience will have observed in very rare cases extraordinary retrogressions of advanced breast tumours with widely spread metastases, and more often delays in the development, which it is difficult to explain as results of therapeutic interventions, and where one is inclined to accept intrinsic biological processes antagonistic to the life and further development of the tumour cells.

If we accept these basic facts, our next problem is to try to assess more precisely the limits of our therapeutic means, and here we meet considerable obstacles.

If two gardeners arrive at the market, one with five and the other with ten boxes of perfect apples, no one will venture an opinion as to who is the most successful gardener without asking how many apple trees each of them is cultivating. This is, however, just what is done in many cases, where statistics are prepared presenting the therapeutic results from different hospitals and institutions without regard to the total number of patients received in the institutions and suffering from the disease under discussion.

Winter (1902) fifty years ago proposed a programme as to how our therapeutic results ought to be presented. These principles have been accepted by the gynecologists co-operating in the League of Nations Health Organization. Haagensen (1949) urged a similar attitude in the statistical presentation of " cures " and survival of sufferers from breast carcinoma, claiming that the absolute or over-all cure rate only is significant, a standpoint strongly supported by McWhirter (1949). The absolute or over-all cure rate is the number of patients alive and symptom-free out of the total number of patients received in the hospital and suffering from the disease under investigation. All dead are counted as victims of said disease, regardless of the real cause of death. Even if this strict attitude is adopted, we meet, however, with factors which influence the statistics. Firstly, the general cancer consciousness of the society, which to a certain degree will influence the relative number of early and late cases. Secondly, social and racial factors, which may influence the soil of the cancer cells, and thirdly, the relative number of cases with moderate and with high malignancy, as shown by Bloom (1950). Kreyberg (1952) in a survey of lung cancer in Norway attempted to show that the difference in the sex distribution of this form of cancer in Norway, as compared to England and Wales, may be explained by the difference in the relative number of the different histological types. A similar situation may hold good for breast carcinoma as well. It is probable, therefore, that besides a plea for the use of absolute cure rates and tumour stages already in use, also a correction for tumour types and grades should be attempted.

Nielsen (1951) gave as reasonable the following figures as the average chances for a breast carcinoma patient in Scandinavia :

5-year survival rate, symptom free (operable cases), 35–50 per cent.
5-year survival rate, symptom free (absolute), 20–30 per cent.

These figures are valid for a population with a generally fair cancer consciousness and with a rather high standard of the doctors and the hospitals. Individual surgeons and special clinics may present better results, but such material is more or less selected.

The cause of the depressingly low cure rate in breast carcinoma is two-fold : (1) the tumour cells have spread beyond surgical control before the operation is performed and even before the diagnosis is made, and/or (2) the tumour cells are not sufficiently radiosensitive.

This state of affairs has naturally led to a quest for other means of improving the therapeutic results, and as one of those, the importance of " early diagnosis " has been universally stressed and accepted. It may be useful therefore to examine the meaning of the designation " early diagnosis ". In the present paper it is intended to examine different definitions of the term " early diagnosis " commonly used in order to ascertain our position today as regards the criteria for the immediate recognition of a breast carcinoma in a stage where a complete cure is possible, if a proper treatment actually at our disposal is used. Especially will be examined the foundation of a propoganda promising great hopes of cure if the patient arrives for treatment on the basis of an " early diagnosis ".

When a breast carcinoma develops morbid changes have usually been present in the tissue for a shorter or a longer period. Even if the essential change is considered to be a somatic mutation in a single cell, this mutation has its local cause. Further, the tissue is not clinically cancerous before the proliferation has reached a certain extent. From a therapeutic standpoint to-day the breast carcinoma is in its early phase as long as the tumour is localized to a degree that makes a complete cure possible by such a relatively simple local operation as mastectomy. This is the background for the designation " Stage I ", and in the absolute sense mastectomy should by definition in such cases result in 100 per cent cures. If a lower percentage is obtained it is because the tumour is question had already spread further and was no longer in Stage I.

One definition of " early diagnosis " ($p_1$), covering a common usage, may be a diagnosis made when the tumour is still in Stage I.

If we inquire into the means of arriving at an early diagnosis, as stated in $p_1$, we find that the simplest staging is the immediate clinical, whereby the palpating fingers try to ascertain the presence or absence of regional lymph nodes. Nohrman (1949) stated that in his series 28 per cent of the cases clinically diagnosed as Stage I, by subsequent histological examination were shown to be in Stage II or higher. Haagensen (1949) quoted a much higher figure, 45 per cent, as covering his experience. This immediate clinical staging is therefore considered useless for scientific and practical work.

More exact is the histological staging, where the grouping of tumours depends upon the finding, or not, of tumour cells in the local lymph nodes. This staging is also subject to serious errors. Firstly, tumour cells may actually be present in the lymph nodes and the pathologist fails to find them. Secondly, tumour cells may have entered other lymphatic channels, by-passing the axillary lymph nodes. Handley (1952) found metastases in the intrathoracic lymph nodes, without involvement of the regional nodes in 3 per cent of his cases, and Margottini (1952) found the same situation in 5 per cent of his cases. Thirdly, a certain number of tumours develop haematogenous metastases without involving any lymph nodes at all. The discrepancy between the number of tumours histologically classified as Stage I and the number of cures effected in this group, after a proper local operation, shows the degree of failure in the process of staging. This failure is approximately 20–30 per cent. In spite of this serious inaccuracy the histological staging is the method used for grouping of tumours for comparing different therapeutic interventions.

The most accurate staging is the retrospective clinical, taking into consideration the final development of the cancerous process in each individual. But even this staging is not perfect. It has been mentioned that patients supposed to be Stage I eventually are shown to be in Stage II or further. On the other hand, patients believed to be Stage I, though actually in Stage II, may never be recognized as such if radiological treatment cures the patient.

This brief survey shows that the decisive determination of the stage of a certain tumour is neither a direct nor an immediate process. A proper and comparatively certain estimate of the correct stage of a tumour takes from 15 to 20 years. The criteria are mainly obtainable as a result of a retrospective clinical survey.

The definition of " early diagnosis ", as given in $p_1$ is useful in theoretical discussions of biological aspects of the tumour problem and for assessing the cure rates in connection with different therapeutic means and circumstances. If, on the other hand, patients are urged to seek " early diagnosis " in cases of breast cancer, the definition, as given in $p_1$, is useless, because the definition does not give criteria immediately available to recognize a breast cancer still in Stage I.

Another attempt at a useful definition of " early diagnosis " may be tentatively a diagnosis made in immediate sequence to the first symptom from the tumour regardless of the type of symptom ($p_2$). This definition is very often silently admitted as valid, and commonly used.

A great number of students have examined the main symptoms of breast cancer, and have correlated the time lag between the discovery of the first symptom and the commencement of treatment on one side and the curative results on the other. It has, generally, tacitly been accepted that the longer the delay, the poorer the results. This sounds very reasonable and the statement evidently holds good in most individual cases, because most tumours begin as a local proliferation and gradually expand and eventually spread.

Korteweg, however, already in 1880 pointed out the remarkable observation that the groups of breast cancer patients with the longest periods of symptoms showed the best curative results, and in a later paper (1889) advanced the explanation that a natural selection of carcinomas with a relatively low malignancy took place during the delay. Bloom (1950) has ably reviewed this problem and has analysed his own material from this angle. His results are shown in Table I.

TABLE I.—*Duration of Symptoms and Prognosis* (*Bloom*, 1950).

| Duration of symptoms. | Cases. | 5-year survivals. Number. | Per cent. |
|---|---|---|---|
| 6 weeks or less | 101 | 50 | 50 |
| 3 months or less | 184 | 94 | 51 |
| 3–6 months | 125 | 59 | 47 |
| 6–12 months | 118 | 56 | 47 |
| 12 months or more | 105 | 55 | 52 |

Bloom concluded that the survival rate is uniform, no matter how long the history. The explanation is that the groups have not a uniform composition. Bloom showed that in the first groups is a greater number of highly malignant tumours than in the latter, and stated that: "We are thus faced with the fact that by the time a highly malignant growth (Grade III) is discovered by the patient it is, in all probability, too late to eradicate, direct extension and metastases having already taken place."

Kreyberg and Christiansen (1953) have recently studied the same problem, considering the grades of the tumours, and have mainly supported Bloom's conclusion.

This conclusion holds good for groups of cases. In single individual cases it will not hold good. Even the most malignant tumour will have a Stage I, however brief the period may be, and even the lowest grade malignant tumour may one day spread and pass into Stage II, or further.

If we turn from the comparison of different groups of patients with varying time lag, and consider the absolute curative results in the groups with the shortest delay in treatment only, we may collect the figures shown in Table II from recent literature.

TABLE II.—*The Cure Rate in Breast Carcinomas with a Short Delay in Treatment.*

| Author. | Duration of symptoms. | Per cent. | Observation period and type of "cure." |
|---|---|---|---|
| Luff (1932) | 4 weeks maximum | 31 | 4 years survival "in good health." |
| Eggers, de Cholnoky, | 4 " " | 76 | 5 years "arrest." |
| Jessup (1941) | 8 " " | 50 | 5 " " |
| Nohrman (1949) | 2 " " | 55 | 5 years "survival." |
| | 4 " " | 52 | 5 " " |
| Kreyberg, Christiansen (1953) | 4 " " | 62 | 10 years "survival symptom free" (very small tumours only). |

This table shows that even in these groups, where the time lag between diagnosis and commencement of treatment is reduced to the practical minimum, only half of the average patients can count upon a 5 years' survival symptom free. It seems, therefore, that the definition $p_2$ is of rather limited usefulness because a great number of the tumours are not any longer curable and the definition has not contained the criteria necessary to obtain this goal. A limited usefulness of $p_2$ is demonstrated by the fact that some patients, an unknown number, have benefited from the shorter time lag—a question which will be discussed later.

If we analyse the usual symptoms, some of them (a feeling of heaviness, pains, stinging sensation and similar) are rather vague and strictly subjective and little fitted for attempts at earlier detection. But among the symptoms analysed, a lump in the breast is the first in at least three-fourths of all cases, and this symptom is more material, more objective and more fitted for diagnostic research. Here a regular and systematic palpation may facilitate the discovery of comparatively small nodules, but there evidently is a certain lower size limit.

Another attempt at a useful definition may accordingly be: a diagnosis of a very small tumour ($p_3$), in the hope that all such tumours actually are curable.

Kaae (1948) voiced the traditional opinion when he stated that: "It is generally recognized that the size of the tumour is a very important factor in the prognosis, and that the latter is much more favourable for the small tumours than for the large."

If we, however, examine the pertinent literature, we shall discover that the students of this problem are not at all unanimous. Bloom (1950), and Kreyberg and Christiansen (1953) have recently considered this question and from the last mentioned paper the following experience may be quoted. The material consisted of small carcinomas, the size varying from that of a hazel nut to that of a pea or a bean. The number of such small carcinomas was less than 6 per cent of the total number of malignant breast tumours examined. The fate of the patients was followed and the long range survey, ten to twenty year's observation, showed that nearly two-thirds of the patients had metastases when arriving for treatment. More than half of the patients died, or would eventually die from their tumours. Even the very smallest carcinomas, those the size of a pea or a bean, had a fatal outcome in four out of ten cases.

These facts show that our attempt to define " early diagnosis " as a diagnosis of a very small tumour ($p_3$), actually a tumour as small as practically diagnosable, also fails miserably.

Taking into consideration the three commonly used or accepted definitions of " early diagnosis ", $p_1$ fails, because the criteria are not immediately available, and $p_2$ and $p_3$ fail, because the criteria do not enable a patient to recognize a tumour still in a curable stage. Nor is any other definition known, which makes such a recognition possible to-day.

What $p_2$ and $p_3$ give are indications for obtaining the earliest possible diagnosis with our present diagnostic means. An examination of the actual degree of earliness shows that only half of the tumours occurring in a population can, under the best possible conditions, be diagnosed early enough to be cured with our present therapeutic means. The reason is that nearly two-thirds of the tumours have metastasized before the tumour is diagnosable, and only a part of those are curable by surgical and/or radiological intervention.

The situation is shown in a simplified form in Fig. 1. The Grade I tumours are very slowly growing and late metastasizing. Early or late diagnosis is not of very great importance. The Grade III tumours are very quickly growing and very early metastasizing. Most of these tumours are beyond therapeutic control before the primary tumour is diagnosed at all. The Grade II tumours are in an intermediate position, and these are the tumours where the question of earliness in diagnosis is of paramount importance. The number of such tumours is not exactly known, but in large series of carcinomas where a well-known history of development is available, data for a reasonable estimate should be at hand.

Under these circumstances it seems strange that statements may be pronounced, claiming that " in early cases of breast cancer " more than 90 per cent

FIG. 1.—Diagrammatic representation of the downward path of patients with the three grades of carcinoma of the breast, in relation to time and the increasing prominence of their symptoms.

are cured. The word " early " is here evidently used in the same meaning as in our definitions, discussed above, where earliness refers to a diagnosis and a treatment when the tumour is still in a curable stage. Earliness and curability are in this connection synonymous terms. The statement that " early cases " have a high curability is therefore a tautology, saying that curable cases have a high curability. The high percentage of cures is the result of a very strict selection of cases. First all cases proved to belong to Stage II, or further, are excluded. This would leave a curability of approximately 75 per cent for the remaining. Next, larger tumours, and finally especially malignant-looking tumours, are excluded. The high percentage of cures is actually an expression of the analyst's ability in retrospective selection of cases. A further improvement in the statistics may be obtained by a still greater efficiency in the process of selection, not necessarily being an expression of increased chances for the patient at large to survive.

It is not correct and not fair to publish such statistics in a manner that makes the public believe that if the women examine themselves regularly and carefully, and pay a visit to a competent doctor at the first suspicion of a symptom from the breast, their chances of a complete cure is in the vicinity of 90 per cent. The truth is that the chances are somewhere in the vicinity of 50 per cent. Kaae (1948) arrived at the same standpoint, and wrote: "It is indicated by these investigations that a 5-year symptom-free survival rate of about 40 per cent is obtainable in Denmark to-day, and that these results may be improved from 40 to 50 per cent, if all patients see a doctor as soon as they notice the first symptom, and if the doctor makes the correct diagnosis and institutes adequate treatment without delay ".

The present study seems to show that a diagnosis as early as possible is of great importance to a certain, yet unknown, number of individual breast carcinoma patients. As we do not beforehand know who will benefit and who will not, a general plea for "early diagnosis" is fully substantiated, as every individual salvaged is important. We must, however, always have in mind that the benefit is statistically moderate, and we ought to be modest in our claims as to the possibility of materially altering the situation for the breast carcinoma patients by this approach to the problem.

It has finally to be added that the present conclusion is based upon the use of the word "early" as an expression of curability seen on the background of our present diagnostic and therapeutic means. As soon as one or both of these factors are altered, a new appreciation of the situation will have to be made.

### SUMMARY AND CONCLUSION.

After a survey of the facts available, according to which the prospective chances of a breast carcinoma patient are shown to be very far from satisfactory, and according to which likewise the advances in treatment and cures have been shown to be very modest, a semantic analysis has been carried out, regarding the foundation of the claims that early diagnosis is of great importance for obtaining better results.

The analysis has shown that the use of the term "early" refers to a state where a breast carcinoma is curable with our present therapeutic means. The term is not used in the sense of a more or less absolute time unit. Earliness is, actually, here synonymous with curability to-day, and this again, in most cases, means a carcinoma in Stage I.

If we examine the means to diagnose a carcinoma still in Stage I, we will discover that such a diagnosis can with a reasonable certainty be made only after a retrospective clinical survey, lasting some 15 to 20 years.

The analysis further shows that we have no criteria enabling an immediate "eary diagnosis", in the sense required. What we have at our disposal are the criteria for making a diagnosis as early as possible with our present diagnostic means. The efficacy of these criteria leads to an estimated optimal absolute cure rate in the vicinity of 50 per cent.

The failure is caused by the fact that an important number of patients have already developed metastases, beyond therapeutic control, at the moment of the earliest possible diagnosis.

A plea for "early diagnosis" should nevertheless be maintained, because earliness is of importance in a certain restricted number of individual cases.

### REFERENCES.

American Radium Society.—(1948) Transactions, 30th Ann. Meeting, Chicago, *Amer. J. Roentgenol.*, **62**, 311.
BACLESSE, F.—(1949) *Ibid.*, **62**, 311.
BLOOM, H. J. G.—(1950) *Brit. J. Cancer*, **4**, 347.
EGGERS, C., DE CHOLNOKY, T., AND JESSUP, D. S. D.—(1941) *Ann. Surg.*, **113**, 321.
ENGELSTAD, R. BULL.—(1948) *Amer. J. Roentgenol.*, **60**, 116.
HAAGENSEN, C. D.—(1949) *Ibid.*, **62**, 328.
HANDLEY, R. S.—(1952) *Proc. Roy. Soc. Med.*, **45**, 565.
KAAE, S.—(1948) *Acta radiol., Stockh.*, **29**, 475.—(1952) *Ibid.*, Suppl. 98.
KORTEWEG, J. A.—(1880) *Ned. Tijdschr. Geneesk* **24** (1) : 121.—(1889) *Langenbeck's Archiv* **38**, Heft 4.
KREYBERG, L.—(1938) *Norsk Mag. Laegevidensk.*, 721.—(1952) *Brit. J. Cancer*, **6**, 112.
*Idem* AND CHRISTIANSEN, T.—(1953) *Ibid.*, **7**, 37.
LUFF, A. P.—(1932) *Brit. med. J.* i : 897.
McWIRTHER, R.—(1949) *Amer. J. Roentgenol.*, **62**, 335.
MARGOTTINI, M.—(1952) *Acta Un. int. Cancr.*, **8**, 176.
NIELSEN, J.—(1951) See Northern Surgical Assoc., p. 211.
NOHRMAN, B.—(1949) *Acta radiol., Stockh.*, Suppl. 77.
Northern Surgical Assoc.—(1951) Transactions, 25th Meeting. Copenhagen: E. Munksgaard, 1951.
WINTER, G.—(1902) *Zbl. Gynäk.*, **26**, 81.